# Tuberculosis Outcomes in Papua, Indonesia: The Relationship with Different Body Mass Index Characteristics between Papuan and Non-Papuan Ethnic Groups

**DOI:** 10.1371/journal.pone.0076077

**Published:** 2013-09-27

**Authors:** Enny Kenangalem, Govert Waramori, Gysje J. Pontororing, Sandjaja, Emiliana Tjitra, Graeme Maguire, Paul M. Kelly, Nicholas M. Anstey, Anna P. Ralph

**Affiliations:** 1 Menzies School of Health Research-National Institute of Health Research and Development Research Program, Timika, Papua Province, Indonesia; 2 District Health Authority, Timika, Papua Province, Indonesia; 3 Public Health and Malaria Control Department, PT Freeport Indonesia, Timika, Papua Province, Indonesia; 4 National Institute of Health Research and Development, Jakarta, Indonesia; 5 Baker IDI Heart and Diabetes Institute, Alice Springs, Northern Territory, Australia; 6 School of Medicine and Dentistry, James Cook University, Cairns, Queensland, Australia; 7 Population Health Division, ACT Government Health Directorate, Canberra, Australian Capital Territory, Australia; 8 Australian National University Medical School, Canberra, Australian Capital Territory, Australia; 9 Global and Tropical Health Division, Menzies School of Health Research and Charles Darwin University, Darwin, Northern Territory, Australia; 10 Department of Infectious Diseases, Division of Medicine, Royal Darwin Hospital, Darwin, Northern Territory, Australia; Universidade Federal do Acre (Federal University of Acre), Brazil

## Abstract

Weight gain achieved during pulmonary tuberculosis (PTB) treatment is associated with the likelihood of bacteriological treatment success. It is recognised that weight and body mass index (BMI) characteristics differ between ethnic groups in health and illness states. However there has been no prior investigation of how ethnic differences in BMI might influence tuberculosis treatment outcome. Our aim was to investigate predictors of microbiological response to PTB treatment at the Tuberculosis Clinic in Timika, Papua Province, Indonesia and specifically, to determine the contribution of ethnicity. The population comprises two distinct ethnic groups - Asian (Non-Papuan) and Melanesian (Papuan). We conducted a prospective study of adults with smear-positive PTB. Treatment outcomes were 1- and 2-month sputum culture and time to microscopy conversion. Clinical measures included weight, BMI, chest radiograph, pulmonary function including forced expiratory volume in 1 second (FEV_1_) and haemoglobin. One hundred eighty six participants (83 Papuan, 103 non-Papuan Indonesians) were enrolled. At baseline, Papuans had higher mean weight and BMI than non-Papuans (50.0 kg versus 46.9 kg, p = 0.006 and 20.0 kg/m2 versus 18.7 kg/m2, p = 0.001 respectively). This was despite having lower mean haemoglobin (11.3 vs 13.1 g/dL, p<0.0001), higher smoking and HIV rates (37% vs 21%, p = 0.02 and 20% vs 5%, p = 0.01 respectively) and longer median illness duration (3 vs 2 months, p = 0.04), but similar radiological severity (proportion with cavities 55% vs 57%, p = 0.7), sputum smear grade (p = 0.3) and mean % predicted FEV_1_ (63% vs 64%, p = 0.7). By 2 months, Papuans had gained still more weight (mean 5.9 vs 4.2 kg, p = 0.02), and were more likely to have negative sputum culture (49/56 vs 45/67, p = 0.02), in univariable and multivariable analyses controlling for other likely determinants of culture conversion. In conclusion, Papuans had better early microbiological outcome from PTB treatment, which may relate to better preservation of weight and greater early weight gain.

## Introduction

Factors which predict response to pulmonary tuberculosis (PTB) treatment need to be clearly defined and should be taken into account when interpreting the findings of TB clinical trials. Interim microbiological measures including 2-month sputum culture conversion [1], [2], time to sputum smear microscopy conversion [3], [4], or time to detection of *Mycobacterium tuberculosis* in sputum culture [5], [6], can be used to help identify patients at risk of unsuccessful treatment 6-month outcome, or subsequent relapse. Since these measures are imperfect, there is active research into biomarkers which might better indicate an individual's response to treatment, and thereby facilitate early endpoints in trials of comparative regimens [2], [7]. In the meantime however, simple and inexpensive measures can continue to shed light on TB responses and offer insights into disease pathophysiology, particularly in high burden under-resourced settings.

Tuberculosis which is more advanced at the time of treatment initiation is recognised to be reliably associated with delayed clearance of *M. tuberculosis* from sputum; thus the presence of cavitary disease and higher bacillary density in sputum at tuberculosis diagnosis are best recognised to predict microscopy and culture conversion in 2-month sputum samples [5], [6], [8]–[11]. Less well understood are the host and organism predictors of outcome, although studies have variously identified older age [5], [8], [9], [11], smoking [5], [6], [9], diabetes [11], male sex [9] and W-Beijing genotype [6] as predictors of slower microbiological response.

In addition to these outcome predictors measurable at baseline, the amount of weight gain achieved by tuberculosis patients during treatment also appears to be associated with microbiological response [12], [13]. Weight gain of ≤5% during the first 2 months of tuberculosis treatment is associated with an increased relapse risk [12], and those experiencing weight loss or poor weight gain are more likely to have unsuccessful 6-month outcomes [14]–[17]. Weight gain is therefore considered an important outcome measure in clinical trials of nutritional interventions in tuberculosis [18], [19]. There is a bi-directional relationship between malnutrition (which impairs cell-mediated immunity), and tuberculosis (which further aggravates weight loss) [20]. Weight is universally measured in TB treatment programs, essentially comprising a free ‘point of care test’, and has been advocated as a simple measure to predict TB treatment outcome [15].

The World Health Organisation (WHO) describes standard body mass index (BMI) cut-points to define categories of nutritional status [21], but these cut-points do not apply equally well across all ethnic groups [22]. Asian populations generally require lower [23], [24], and Melanesians, higher [25], [26] cut-points. BMI cannot distinguish lean from fat mass and is an imprecise surrogate for nutritional status, but is the most practical, inexpensive measure [27].

There is limited evidence pertaining to the influence of ethnicity on responses to tuberculosis treatment. Such studies are generally challenging due to the difficulty in comparing outcomes between different geographical sites. In a population of tuberculosis patients in eastern Indonesia comprising two distinct ethnic groups - Asian (Non-Papuan) and Melanesian (Papuan), who have recognisably different anthropomorphic characteristics, we sought to identify the predictors of microbiological outcome of PTB treatment, and determine the contribution of ethnicity.

## Materials and Methods

This study was performed as part of a clinical trial of adjunctive L-arginine and vitamin D for PTB (clinicaltrials.gov/NCT00677339) [28], [29]. In this study, neither intervention significantly affected the outcome measures [28]. Eligible sequential study participants with PTB who had no previous history of treatment were enrolled at the Tuberculosis clinic in Timika, Papua Province, Indonesia. The TB case notification rate in Timika has been estimated at 311/100 000 [30]. Inclusion criteria included age ≥15 years, sputum smear-positive for acid fast bacilli (AFB), and provision of written informed consent from the participant, or guardian if aged <18. Standard tuberculosis treatment was administered in a directly observed fashion in fixed-dose combination (FDC) tablets containing rifampicin 150 mg, isoniazid 75 mg, pyrazinamide 400 mg and ethambutol 272 mg for 2 months (30–39 kg: 2 tablets daily; 40–54 kg: 3 tablets daily; 55–70 kg: 4 tablets daily; ≥71 kg: 5 tablets daily), then rifampicin and isoniazid for 4 months also in standard weight-adjusted doses [31]. Local healthy controls were eligible if they were aged ≥18 years, gave written informed consent, and had no co-morbidities.

### Ethics statement

The study was approved by the Human Research Ethics Committees of Menzies School of Health Research, Darwin, Australia and the National Institute for Health Research and Development, Jakarta, Indonesia. All participants provided written informed consent.

### Definitions and procedures

Treatment outcome was categorised as cured, completed, failed, died, defaulted or transferred out [4], or dichotomised as successful (cured, completed) or unsuccessful (failed, defaulted, died), as described elsewhere [14]. Chest radiographs, pulmonary function, a 6-minute walk test (6MWT) and a locally adapted St George's Respiratory Questionnaire (SGRQ, in Indonesian) were performed at baseline then at 2 and 6 months. The SGRQ is a respiratory health-related quality of life instrument, shown to be an effective tool in measuring PTB morbidity [32], [33]. Low scores indicate better lung health. We have previously reported on the use of the Indonesian modified SGRQ among TB patients in Timika [33]. Chest x-rays were scored according to a previously-reported method [34]. Pulmonary function (forced vital capacity, FVC and forced expiratory volume in 1 second, FEV_1_) was measured outdoors (for infection control purposes) using a handheld spirometer (MicroLoop®, MicroMedical, UK), with individual-use filtered one-way mouthpieces (Sure-Gard®) suitable for use in smear-positive PTB. Percentage of predicted FEV_1_ was calculated from local normal reference ranges [35]. 6MWT was assessed according to standard procedures [36].

Standing height was measured without shoes to the nearest centimetre using a stadiometer; weight was measured without shoes to the nearest 0.1 kg using an adult balance; BMI was calculated in the standard way: weight (kg) / height (m)^2^. Venous full blood count and haemoglobin concentration were measured using a Sysmex KX-21N™ Automated Hematology Analyzer. HIV antibody testing was as per standard local practice using a rapid point-of-care test (Standard Diagnostics BioLine HIV-1/2 3.0™), with positive results confirmed on 2 additional rapid tests (Abbott Determine™ HIV-1/2 [Inverness Medical], and Oncoprobe™ [PT Oncoprobe Utama].

Sputum microscopy was undertaken at the Timika field laboratory weekly for 2 months then at months 5 and 6. Baseline, 1-month and 2-month sputum cultures were processed at a World Health Organisation-accredited reference laboratory at the University of Indonesia, Jakarta, using the BACTEC® Mycobacterium Growth Indicator Tube (MGIT) 960 system. Sputum culture conversion at 2 months is a standard early measure of TB treatment response [1], [2]. Sputum smear conversion was defined as ≥2 consecutive negative smears without a subsequent positive.

### Statistical methods

Analyses were undertaken using Stata 12.1. Proportions were compared using Chi-squared test, or Fisher's exact test where necessary. Continuous variables were compared using Student's 2-sample T test or Wilcoxon rank sum test depending on their distribution. Associations between sputum culture status and other measures were tested using univariable and multivariable logistic regression models. Multivariable models were created using a backwards stepwise approach, including in the first model all variables shown to be related to culture status in univariable models (p≤0.06), and variables which differed between ethnic groups at baseline (e.g. age, HIV status). Kaplan-Meier survival analysis was used to examine sputum smear conversion time; patient subgroup analyses were performed by Cox regression (proportional-hazards) models and hazard ratios and 95% confidence intervals.

## Results

### Baseline study participant characteristics

**Figure 1:**
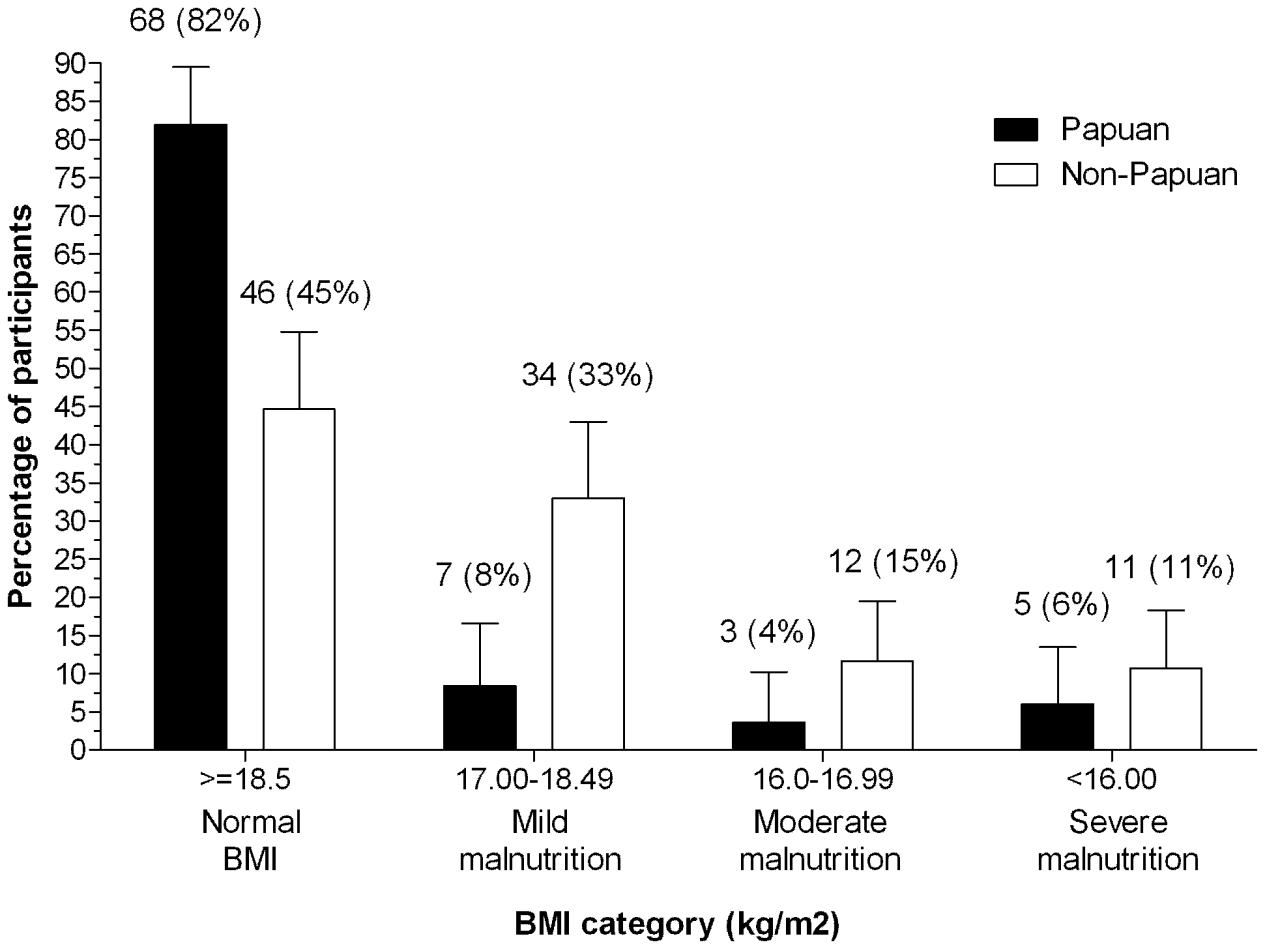
Body mass index at TB diagnosis.

One hundred and eighty six participants (83 Papuan, 103 non-Papuan Indonesians) with PTB who met inclusion criteria were enrolled June 2008 - November 2009 and followed up for 6 months. Participants' characteristics showing differences between the two ethnic groups are provided in Table 1. There were two instances of multidrug-resistant (MDR)-TB identified, both in non-Papuan participants. Papuans with PTB were younger than non-Papuans, were more likely to smoke, be HIV positive, report longer illness duration prior to diagnosis, and have lower haemoglobin (Table 1). Papuan males had significantly higher baseline bodyweight and BMI, and were able to walk further, than non-Papuans. Most Papuans (82%) but less than half of the non-Papuans (45%) had a BMI considered ‘normal’ according to the standard definition (≥18.5 mg/kg^2^) (Table 1; Figure 1). Similar differences were seen in women with PTB, but their numbers were smaller and the differences were not statistically significant. No such differences were observed between ethnic groups in 38 healthy controls (Table 1).

**Table 1:** Characteristics of study participants.

	Papuans	Non-Papuans	p value
**TUBERCULOSIS PATIENTS AT DIAGNOSIS: Number**	83	103	
**Age in years: median (range)**	26 (21–35)	30 (24–38)	**0.005**
**Female: no. (%)**	26 (31%)	33 (34%)	0.7
**HIV positive: no./no.tested (%)**	14/70 (20%)	4/74 (5%)	**0.01**
**Current smoker: no. (%)**	31 (37%)	22 (21%)	**0.02**
**Height (m): mean (SD)**	1.58 (0.71)	1.58 (0.08)	0.8
**Weight (kg): mean (SD)**			
All	50.0 (6.9)	46.9 (7.9)	**0.006**
Female	44.4 (6.8)	42.5 (8.7)	0.4
Male	52.5 (5.3)	49.2 (6.5)	**0.002**
**Body mass index (kg/m^2^): mean (SD)**			
All	20.0 (2.4)	18.7 (2.8)	0.001
Female	19.1 (3.1)	18.7 (3.8)	0.6
Male	20.4 (1.7)	18.7 (2.2)	<0.0001
**Fixed-dose combination recommended starting dose: no. (%)**			
2 tablets (30–39 kg)	6 (7%)	17 (17%)	**0.09**
3 tablets (40–54 kg)	57 (69%)	69 (67%)	
4 tablets (55–70 kg)	20 (24%)	16 (16%)	
5 tablets (≥71 kg)	0 (0%)	1 (1%)	
**Illness duration (months): median (IQR)**	3 (1–5)	2 (1–4)	**0.04**
**Haemoptysis: no. (%)**	22 (27%)	35 (34%)	0.3
**%Predicted FEV_1_: mean (SD)**	63 (20)	64 (19)	0.7
**St George**'**s Respiratory Questionnaire total score: median (IQR)**	40.6 (27.2–55.8)	41.4 (23.9–57.5)	0.8
**6-minute walk test: median (IQR)**			
Female	379 (303–425)	369 (332–410)	0.6
Male	454 (396–489)	419 (335–450)	0.04
**Chest radiograph**			
Cavity disease: no.(%)	41/75 (55%)	47/82 (57%)	0.7
% lung affected: median (IQR)	45 (23–71)	36.5 (21–54)	0.3
X-ray score: median (IQR)	73 (29–100)	63 (36–88)	0.5
**Sputum acid fast bacilli density: no.(%)**			
0 or Scanty	25 (30)	24 (23)	0.3
1+	26 (31)	26 (25)	
2+	17 (20)	33 (32)	
3+	15 (18)	20 (19)	
**Haemoglobin (g/dL): mean (SD)**			
Female	10.4 (1.7)	12.1 (2.0)	**0.0009**
Male	11.6 (1.6)	13.6 (2.3)	**<0.0001**
***M. tuberculosis*** ** identified from sputum at diagnosis**	68/75 (91%)	88/97 (91%)	1.0
Resistant to rifampicin and isoniazid	0	2/88 (2.3%)	
**TB PATIENTS AT TREATMENT COMPLETION**			
**Weight (kg): mean (SD)**			
All	54.4 (7.1)	52.8 (9.0)	0.3
Female	50.1 (8.4)	48.2 (10.1)	0.5
Male	56.7 (5.0)	55.1 (7.5)	0.3
**Body mass index (kg/m^2^): mean (SD)**			
All	22.1 (2.8)	21.1 (3.3)	0.06
Female	21.8 (3.9)	21.2 (4.3)	0.7
Male	22.3 (2.0)	21.0 (2.6)	**0.02**
**Xray score**			
Cavity disease: no.(%)	6/38 (15%)	8/39 (21%)	0.4
% lung affected: median (IQR)	6 (2–14)	11.5 (4–20)	0.06
X-ray score: median (IQR)	6 (2–15)	12.5 (4–20.5)	0.08
**HEALTHY VOLUNTEERS: Number**	19	19	
**Female: no. (%)**	6 (32%)	3 (16%)	0.2
**Weight (kg): mean (SD)**	61.6 (10.4)	63.4 (9.9)	0.6
**Body mass index (kg/m^2^): mean (SD)**	24.2 (4.1)	24.4 (3.7)	0.9

Among the 18 HIV positive participants, CD4 T cell counts were measurable in 6 individuals (median 318 cells/µL, range 18–739), and only 2 were able to receive antiretroviral therapy during TB treatment, one from before TB diagnosis, the other from week 20, as described elsewhere [37]. Clinical features additional to the findings consistent with PTB included cervical lymphadenopathy in 1 person and oro-oesoephageal candididasis in 4 people.

The TB medication dosage received by Papuans was not statistically significantly different from that received by non-Papuans, since most people of both ethnic groups were in the 40–54 kg band, for whom 3 FDC tablets daily is recommended (Table 1). There were no significant ethnic differences in radiological severity (presence of cavitary disease, extent of lung involvement or overall score), proportion of people with haemoptysis, pulmonary function, or the subjective quality of life measure (SGRQ).

### Outcomes

**Figure 2:**
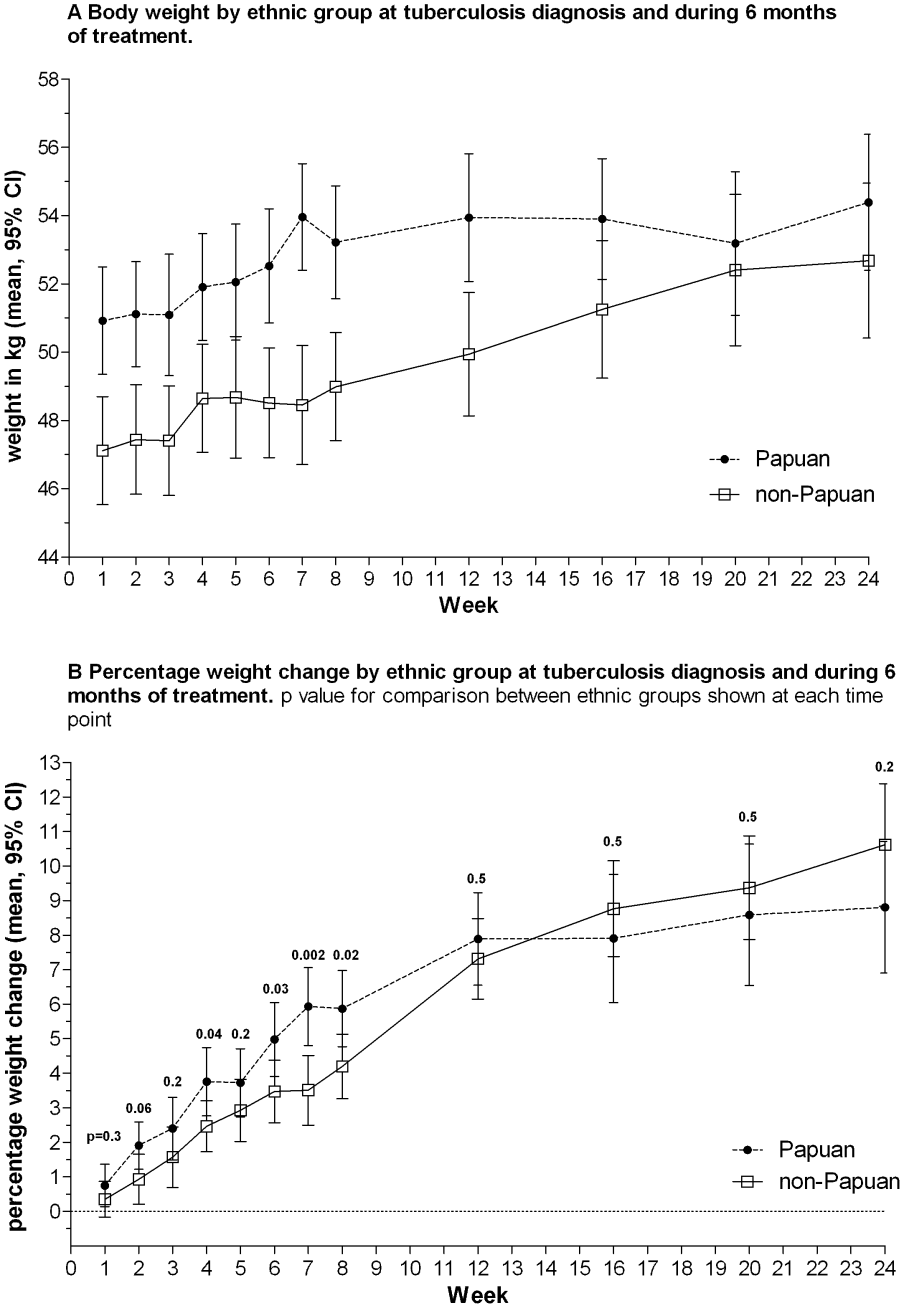
Weight according to ethnic group. A. Body weight by ethnic group at tuberculosis diagnosis and during 6 months of treatment. B. Percentage weight change by ethnic group at tuberculosis diagnosis and during 6 months of treatment. p value for comparison between ethnic groups shown at each time point

**Figure 3:**
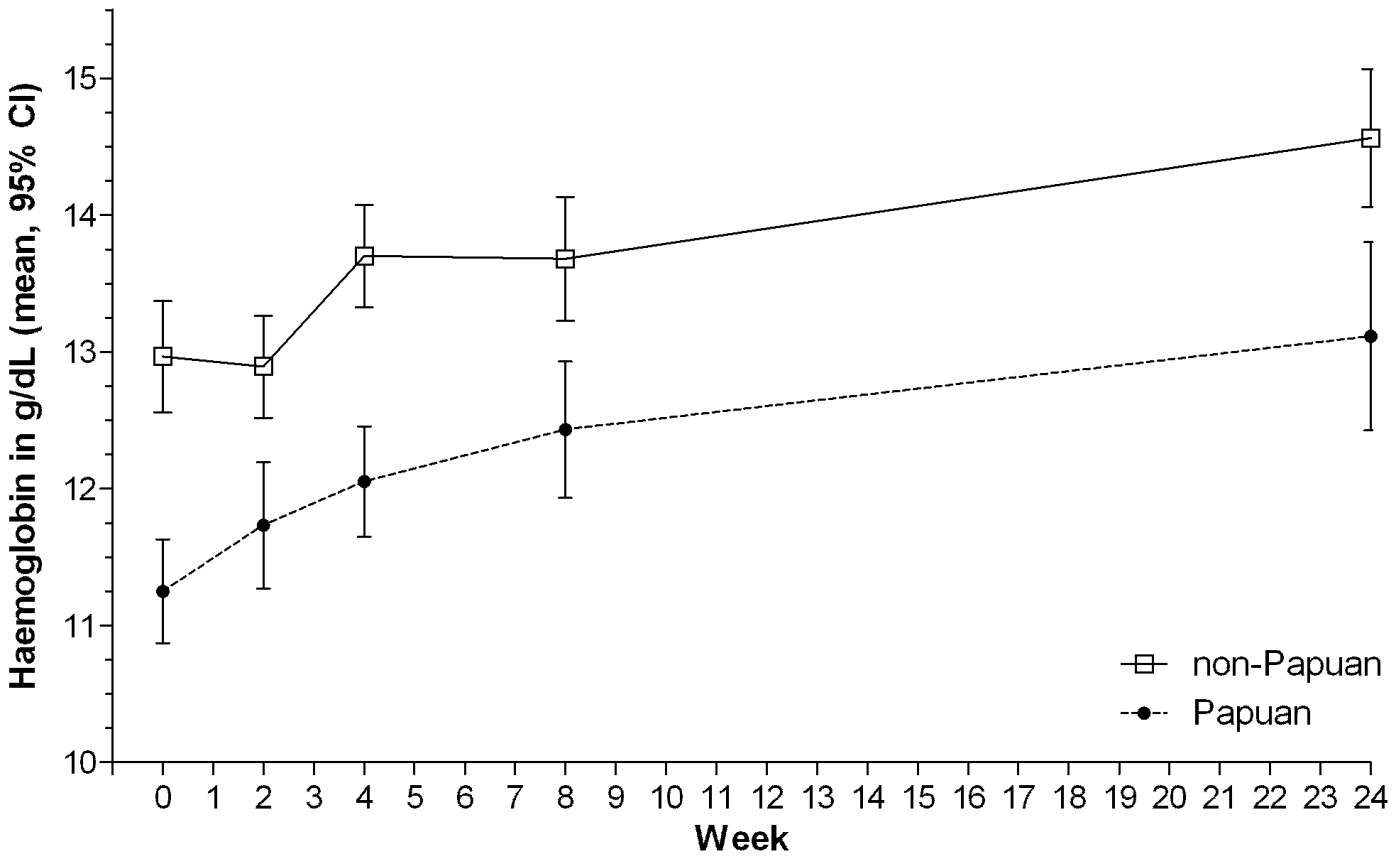
Haemoglobin by ethnic group at tuberculosis diagnosis and during 6 months of treatment.

As well as having greater weight and BMI at diagnosis (Table 1, Figures 1 and 2), Papuans showed larger early increases in weight than non-Papuans, which later plateaued (Figures 2A and B). Non-Papuans gained weight steadily throughout treatment, such that by treatment completion, the gap in weight between the ethnic groups had closed (at 6 months, mean weight in Papuans: 54.4 kg, versus Non-Papuans: 52.8 kg, p = 0.3). Haemoglobin was lower in Papuans throughout the study (Table 1 and Figure 3). At treatment completion, there was a trend toward higher chest X-ray scores in Papuans, but the difference was not statistically significant, and less than half the participants had a 6-month x-ray reported (Table 1). Other clinical measures (pulmonary function measures, 6MWT, SGRQ) did not differ between ethnic groups at 6 months (data not shown).

**Figure 4:**
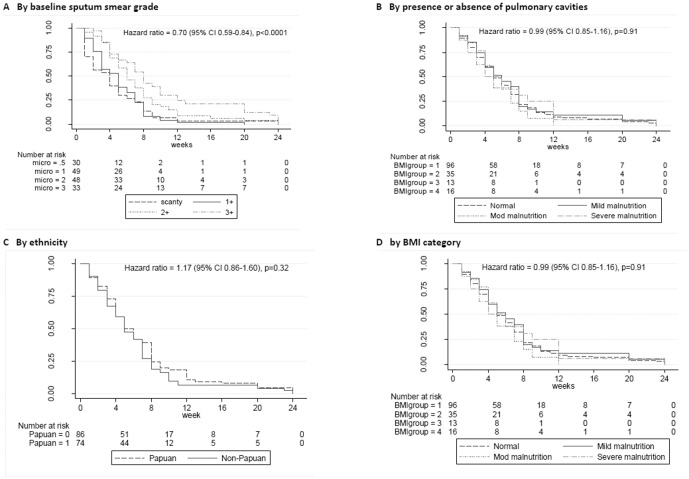
Kaplan-Meier survival curve showing probability of sputum smear conversion. A. by baseline sputum smear grade. B. by presence or absence of pulmonary cavities. C. by ethnicity. D. by BMI category.

The median time to smear conversion was 5 weeks (IQR 2–8). Baseline smear grade and cavitary status, but no other variables, predicted time to smear conversion (Figure 4A–D).

The proportions of study participants achieving negative sputum cultures were 62% (93/151) at 1 month and 78% (94/120) at 2 months. Culture conversion likelihood at months 1 and 2 were closely related (p = 0.007), but determinants of culture conversion at these time points differed. In univariable analyses, baseline sputum smear grade, pulmonary function, total x-ray score, presence of cavities and illness duration all predicted 1-month culture conversion (Table 2). In multivariable analyses, both x-ray score (p = 0.002) and sputum smear grade (p = 0.059) remained as significant predictors (Table 2). By 2 months, baseline sputum smear grade remained an important determinant, but ethnicity and weight also showed significant associations with culture conversion; ethnicity and baseline sputum AFB grade remained significant in the multivariable model (Table 2). Culture conversion had been achieved at 2 months by 49/56 (88%) of Papuans, and 45/64 (70%) of non-Papuans (p = 0.02). Multi-drug resistance was detected in only 2 people and was not significantly associated with outcome: one patient with MDR-TB was transferred during week 2 and hence no follow up samples were available, and sputum culture status in the other was unavailable at 1 month but positive at 2 months.

**Table 2:** Associations between sputum culture conversion and clinical variables in univariable and multivariable analyses (p valus <0.05 shown in bold).

	Independent variable: 1-month sputum culture		Independent variable: 2-month sputum culture	
	(1 = positive, 0 = negative)		(1 = positive, 0 = negative)	
Dependent variable	Univariable odds ratio (95% CI), p value	Mulitvariable	Univariable	Mulitvariable
**Female (vs male)**	0.77 (0.37–1.60), p = 0.49	NS	1.25 (0.50–3.13), p = 0.64	NS
**Age (years)**	1.00 (0.97–1.03), p = 0.96	NS	1.02 (0.99–1.06), p = 0.22	NS
**Weight (kg)**	0.98 (0.93–1.02), p = 0.29	NS	0.93 (0.87–0.99), **p = 0.03**	NS
**Current smoker**	0.47 (0.22–1.02), p = 0.06	NS	0.87 (0.33–2.30), p = 0.78	NS
**Papuan (vs Non-Papuan)**	1.16 (0.60–2.24), p = 0.65	NS	0.34 (0.13–0.88), **p = 0.03**	0.36 (0.14–0.97), **p = 0.04**
**HIV+ (vs HIV-)**	0.98 (0.35–2.73), p = 0.97	NS	0.24 (0.03–1.95), p = 0.18	NS
**Sputum acid fast bacillus density (grade)**	1.83 (1.29–2.60), **p = 0.001**	1.46 (0.99–2.12), p = 0.06	1.92 (1.21–3.05), **p = 0.005**	0.87 (1.17–3.00), **p = 0.009**
**Cavitary disease**	3.45 (1.64–7.26), **p = 0.001**	NS	2.26 (0.85–6.01), p = 0.10	NS
**Xray score (units)**	1.02 (1.01–1.03), **p<0.0001**	1.02 (1.01–1.03), **p = 0.002**	1.01 (1.00–1.02), p = 0.12	NS
**FVC (L)**	0.68 (0.43–1.06), p = 0.09	NS	0.96 (0.54–1.69), p = 0.88	NS
**FEV1 (L)**	0.57 (0.34–0.96), **p = 0.04**	NS	0.94 (0.49–1.80), p = 0.86	NS
**% Predicted FEV1 (L)**	10.97 (0.96–0.99), **p = 0.006**	NS	1.00 (0.98–1.03), p = 0.62	NS
**Haemoglobin (g/dL)**	0.91 (078–1.06), p = 0.22	NS	1.03 (0.80–1.33), p = 0.81	NS
**White cell count**	1.16 (1.04–1.30), p = **0.008**	NS	1.10 (0.95–1.28), p = 0.22	NS

Seven participants were transferred elsewhere for treatment completion. In the remaining 179, 6-month TB treatment outcome was successful in 146 (81.6%) and unsuccessful in 33 (18.4%) (Table 3). Two people died and two failed. No significant associations could be identified between microbiological outcomes (4 or 8-week culture or time to microscopy conversion) or other patient variables (e.g. ethnicity, sex, HIV or smoking status, presence of drug resistance and presence of cavitary lung disease on x-ray) and 6-month TB outcome category. However the 4 people who died or failed were on average 10 years older (mean age 40.8 years) than the 146 participants with a successful outcome (30.6 years), p = 0.08.

**Table 3:** Six month outcome.

Outcome	N (%)	Culture positive at week 4: n(%)	Culture positive at week 8: n(%)
Cured	118 (63)	37/105 (35)	15/89 (17)
Completed	28 (15)	7/20 (35)	4/16 (25)
Failed	2 (1)	1/1 (100)	2/2 (100)
Defaulted	29 (16)	10/21 (48)	3/10 (30)
Died	2 (1)	1/2 (50)	0/1 (0)
Transferred	7 (4)	2/2 (100)	2/2 (100)
TOTAL	186	58 (31.2)	26 (14.0)

## Discussion

We have demonstrated that TB patients of Papuan ethnicity in eastern Indonesia are more likely to achieve sputum culture clearance at 2 months, and maintain higher body weight despite having similar or worse disease severity than non-Papuan Indonesians. Weight maintenance and greater early percentage weight gains after commencing treatment may partly explain the early outcome advantage seen in the Papuan ethnic group. The difference between ethnic groups decreased during the continuation phase of treatment, with no difference in likelihood of treatment success seen at 6 months, and no persisting difference in weight at treatment completion; however, BMI remained slightly higher among Papuan than non-Papuan males, and there was a suggestion of possible better radiological outcomes in Papuans. The Papuans in this study were slightly younger than non-Papuans. Younger age (e.g. ≤65 versus >65 years) has been associated with more rapid smear or culture conversion in some studies [8], [9], [11]. However, age was not a significant predictor of microbiological outcome in this study, and the association between ethnicity and outcome remained significant when accounting for age (Table 2).

Differences in BMI, and in the relationship between BMI and body fat stores, are recognised in healthy populations of different ethnicity [23]–[26], [38]. Here the difference appears to be particularly accentuated in the setting of pulmonary TB, being less evident by treatment completion, and not present in the healthy controls. Greater weight loss in the non-Papuans was not attributable to greater disease severity in this group, on the basis of radiological grade, AFB density or other measures of severity at diagnosis. In fact taking into account HIV and smoking rates, reported illness duration prior to treatment commencement, and haemoglobin, Papuans might have been expected to have been more unwell, and have lower BMI, in contrast to our findings.

The pathogenesis of weight loss in TB is multi-factorial. Expression of tumour necrosis factor-α (TNF-α, ‘cachexin’) is an important component of the human antimycobacterial immune response [39], but is also thought to be responsible in part for TB-related cachexia [40], [41]. ‘Anabolic block’, whereby utilization of amino acids for protein synthesis may be impaired by pro-inflammatory cytokines, has also been proposed as a contributor to TB-related weight loss [42]. Disease-related loss of appetite also occurs. The extent of weight loss correlates with radiological severity in TB in some studies [43]. Our findings are hypothesis-generating: for example, it is possible that Papuans express less TNF-α in response to TB infection, or have different dietary habits in the context of illness. We were unable to systematically evaluate dietary intake in all study participants prior to and during treatment. Differences in muscle or bone mass may also contribute, but not to a large extent, given the lesser differences noted in weight at treatment completion or in the healthy controls. Anti-TB medication dosing is weight-based, but higher TB drug dosing does not appear to explain the ethnic differences, since the recommended FDC dosing is the same between 40 and 54 kg, and Papuans did not receive higher doses overall than non-Papuans.

The higher HIV rate in Papuans than non-Papuans in patients included in this dataset has been reported previously [37]. The lower haemoglobin among Papuans is likely to be due to a range of factors and cannot be considered a reliable marker of nutritional status in this environment: in addition to the TB-related anaemia of chronic disease which improved with institution of TB treatment (Figure 3), the ethnic disparity may relate to differential rates between these groups of malaria, iron deficiency, intestinal helminth infections or haemoglobinopathies [44]. Disparities in haemoglobin were not related to proportions of females, which were similar among the Papuans and non-Papuan groups in this study (31 vs 34%, Table 1).

Limitations of the study include that additional measures of nutritional status, such as skinfold thicknesses or dual energy X-ray absorptiometry, were not undertaken, but these are not usually performed as part of routine TB care, whereas weight is universally included in the baseline and follow-up assessments of TB patients. Our number of healthy volunteers was small, but the trend in weight /BMI seen in TB patients during recovery supports the findings in the healthy control group.

Our findings on the importance of baseline smear grade and cavitary status in predicting early TB microbiological outcomes concur with many previous studies [6], [8]–[11]. However of note, we also found that the predictors of TB outcome varied at different time points. Culture conversion at 1 month was associated in univariable analyses with measures of baseline disease severity, whereas host factors (weight and ethnicity) appeared to become more important by 2 months. Lack of an association between the 6-month outcome and earlier microbiological results is not unexpected since an unsuccessful outcome (death or default) occurred prior to being able to collect 1- or 2-month sputum specimens in some instances, and numbers with unsuccessful outcomes were small (Table 3). 6-month outcome categories are appropriate for programmatic monitoring, but provide minimal information of discriminatory value about patient clinical outcomes. This emphasises that TB biomarkers which could provide better measures of treatment response than standard 6-month outcome categories are required, and that alternative measures of illness impact are needed to assess clinically relevant measures of TB outcome such as residual pulmonary disability [33].

## Conclusions

In conclusion, ethnicity accounts for differences in response to tuberculosis treatment in people inhabiting the same environment and receiving the same care through a single tuberculosis clinic. Despite the widespread use of weight or BMI to measure nutritional status and response to treatment in tuberculosis, this study highlights potential flaws in comparing these measures across ethnic groups. ‘Normal’ BMI (≥18.5 kg/m^2^) might underestimate disease severity in Papuans, and cut-points for BMI categories should be considered as a guide only. When interpreting the results of TB research in which absolute or percentage changes in weight are measured, the potential for intrinsic differences between ethnic groups needs to be acknowledged.

## References

[1] Mitchison DA (1993) Assessment of new sterilizing drugs for treating pulmonary tuberculosis by culture at 2 months. Am Rev Respir Dis 147: 1062–1063. 8466107 10.1164/ajrccm/147.4.1062

[2] Wallis RS, Doherty TM, Onyebujoh P, Vahedi M, Laang H, et al (2009) Biomarkers for tuberculosis disease activity, cure, and relapse. Lancet Infect Dis 9: 162–172. 19246020 10.1016/S1473-3099(09)70042-8

[3] Rieder HL (1996) Sputum smear conversion during directly observed treatment for tuberculosis. Tuber Lung Dis 77: 124–129. 8762846 10.1016/s0962-8479(96)90026-x

[4] World Health Organisation (2010) Treatment of tuberculosis Guidelines Fourth edition WHO/HTM/TB/2009.420 WHO, Geneva, Switzerland

[5] Dorman SE, Johnson JL, Goldberg S, Muzanye G, Padayatchi N, et al (2009) Substitution of moxifloxacin for isoniazid during intensive phase treatment of pulmonary tuberculosis. Am J Respir Crit Care Med 180: 273–280. 19406981 10.1164/rccm.200901-0078OC

[6] Visser ME, Stead MC, Walzl G, Warren R, Schomaker M, et al (2012) Baseline predictors of sputum culture conversion in pulmonary tuberculosis: importance of cavities, smoking, time to detection and W-Beijing genotype. PLoS ONE 7: e29588. 22238625 10.1371/journal.pone.0029588 PMC3251579

[7] Maertzdorf J, Weiner J 3rd, Kaufmann SH (2012) Enabling biomarkers for tuberculosis control. Int J Tuberc Lung Dis 16: 1140–1148. 22871324 10.5588/ijtld.12.0246

[8] Dominguez-Castellano A, Muniain MA, Rodriguez-Bano J, Garcia M, Rios MJ, et al (2003) Factors associated with time to sputum smear conversion in active pulmonary tuberculosis. Int J Tuberc Lung Dis 7: 432–438. 12757043

[9] Guler M, Unsal E, Dursun B, Aydln O, Capan N (2007) Factors influencing sputum smear and culture conversion time among patients with new case pulmonary tuberculosis. Int J Clin Pract 61: 231–235. 17166185 10.1111/j.1742-1241.2006.01131.x

[10] Telzak EE, Fazal BA, Pollard CL, Turett GS, Justman JE, et al (1997) Factors influencing time to sputum conversion among patients with smear-positive pulmonary tuberculosis. Clin Infect Dis 25: 666–670. 9314458 10.1086/513772

[11] Wang JY, Lee LN, Yu CJ, Chien YJ, Yang PC (2009) Factors influencing time to smear conversion in patients with smear-positive pulmonary tuberculosis. Respirology 14: 1012–1019. 19659516 10.1111/j.1440-1843.2009.01598.x

[12] Khan A, Sterling TR, Reves R, Vernon A, Horsburgh CR (2006) Lack of weight gain and relapse risk in a large tuberculosis treatment trial. Am J Respir Crit Care Med 174: 344–348. 16709935 10.1164/rccm.200511-1834OC

[13] Yew WW, Leung CC (2006) Prognostic significance of early weight gain in underweight patients with tuberculosis. Am J Respir Crit Care Med 174: 236–237. 16864715 10.1164/rccm.200605-669ED

[14] Hoa NB, Lauritsen JM, Rieder HL (2012) Changes in body weight and tuberculosis treatment outcome in Viet Nam. Int J Tuberc Lung Dis 1: 61–66. 10.5588/ijtld.12.0369 23146565

[15] Bernabe-Ortiz A, Carcamo CP, Sanchez JF, Rios J (2011) Weight variation over time and its association with tuberculosis treatment outcome: a longitudinal analysis. PLoS ONE 6: e18474. 21494617 10.1371/journal.pone.0018474 PMC3072983

[16] Vasantha M, Gopi PG, Subramani R (2009) Weight gain in patients with tuberculosis treated under directly observed treatment short-course (DOTS). Indian J Tuberc 56: 5–9. 19402266

[17] Krapp F, Veliz JC, Cornejo E, Gotuzzo E, Seas C (2008) Bodyweight gain to predict treatment outcome in patients with pulmonary tuberculosis in Peru. Int J Tuberc Lung Dis 12: 1153–1159. 18812045

[18] Martins N, Morris P, Kelly PM (2009) Food incentives to improve completion of tuberculosis treatment: randomised controlled trial in Dili, Timor-Leste. BMJ 339: b4248. 19858174 10.1136/bmj.b4248 PMC2767482

[19] Sinclair D, Abba K, Grobler L, Sudarsanam TD (2011) Nutritional supplements for people being treated for active tuberculosis. Cochrane Database Syst Rev: CD006086. 10.1002/14651858.CD006086.pub2 18843702

[20] Cegielski JP, McMurray DN (2004) The relationship between malnutrition and tuberculosis: evidence from studies in humans and experimental animals. Int J Tuberc Lung Dis 8: 286–298. 15139466

[21] World Health Organization (1999) Management of severe malnutrition: a manual for physicians and other senior health workers. In: WHO Library Cataloguing in Publication Data G, editor. Geneva.

[22] World Health Organisation (2006) Global Database on body mass index: Body mass index classification. http://apps.who.int/bmi/index.jsp?introPage=intro_3.html.vAccessed 2012 Dec 5.

[23] World Health Organisaion Expert Consultation (2004) Appropriate body-mass index for Asian populations and its implications for policy and intervention strategies. Lancet 363: 157–163. 14726171 10.1016/S0140-6736(03)15268-3

[24] Chen X, Wang Y (2010) Commentary: optimal body mass index cut points. Int J Epidemiol 39: 1045–1047. 20488882 10.1093/ije/dyq081 PMC2929354

[25] Brian G, Ramke J, Page A, Maher L, Szetu J, et al (2011) The association of diabetes and BMI among Melanesian and Indian Fijians aged >/ = 40 years. Br J Nutr 105: 1539–1545. 21255475 10.1017/S0007114510005258

[26] Brian G, Ramke J, Maher L, Page A, Fischer-Harder K, et al (2011) Body mass index among Melanesian and Indian Fijians aged >/ = 40 years living in Fiji. Asia Pac J Public Health 23: 34–43. 21169598 10.1177/1010539510390665

[27] Gallagher D, Visser M, Sepulveda D, Pierson RN, Harris T, et al (1996) How useful is body mass index for comparison of body fatness across age, sex, and ethnic groups? Am J Epidemiol 143: 228–239. 8561156 10.1093/oxfordjournals.aje.a008733

[28] Ralph AP, Waramori G, Pontororing GJ, Kenangalem E, Wiguna A, et al.. (2013) L-arginine and Vitamin D adjunctive therapies in pulmonary tuberculosis: a randomised, double-blind, placebo-controlled trial. PLoS ONE accepted. 10.1371/journal.pone.0070032 PMC3743888 23967066

[29] Ralph AP, Yeo TW, Salome CM, Waramori G, Pontororing GJ, et al.. (2013) Impaired Pulmonary Nitric Oxide Bioavailability in Pulmonary Tuberculosis: Association with Disease Severity and Delayed Mycobacterial Clearance with Treatment. J Infect Dis. 10.1093/infdis/jit248 PMC3719909 23737604

[30] Ardian M, Meokbun E, Siburian L, Malonda E, Waramori G, et al (2007) A public-private partnership for TB control in Timika, Papua Province, Indonesia. Int J Tuberc Lung Dis 11: 1101–1107. 17945067

[31] World Health Organisation (2002) Operational guide for the for national tuberculosis programs on the introduction and use of fixed-dose combination drugs. WHO/CDS/TB/2002.308.

[32] Pasipanodya JG, Miller TL, Vecino M, Munguia G, Bae S, et al (2007) Using the St. George respiratory questionnaire to ascertain health quality in persons with treated pulmonary tuberculosis. Chest 132: 1591–1598. 17890471 10.1378/chest.07-0755

[33] Maguire GP, Anstey NM, Ardian M, Waramori G, Tjitra E, et al (2009) Pulmonary tuberculosis, impaired lung function, disability and quality of life in a high-burden setting. Int J Tuberc Lung Dis 13: 1500–1506. 19919767

[34] Ralph AP, Ardian M, Wiguna A, Maguire GP, Becker NG, et al (2010) A simple, valid, numerical score for grading chest x-ray severity in adult smear-positive pulmonary tuberculosis. Thorax 65: 863–869. 20861290 10.1136/thx.2010.136242

[35] Handojo T, Anstey N, Kelly P, Pain M, Kenangalem E, et al (2006) Normal spirometry, gas transfer and lung volume values in Papua, Indonesia. Southeast Asian J Trop Med Public Health 37: 571–577. 17120982

[36] American Thoracic Society (2002) ATS statement: guidelines for the six-minute walk test. Am J Respir Crit Care Med 166: 111–117. 12091180 10.1164/ajrccm.166.1.at1102

[37] Pontororing GJ, Kenangalem E, Lolong DB, Waramori G, Sandjaja, et al (2010) The burden and treatment of HIV in tuberculosis patients in Papua Province, Indonesia: a prospective observational study. BMC Infect Dis 10: 362. 21605474 10.1186/1471-2334-10-362 PMC3022835

[38] World Health Organisation (2006) Global Database on body mass index: Body mass index classification. http://apps.who.int/bmi/index.jsp?introPage=intro_3.html.

[39] Flynn JL, Goldstein MM, Chan J, Triebold KJ, Pfeffer K, et al (1995) Tumor necrosis factor-alpha is required in the protective immune response against Mycobacterium tuberculosis in mice. Immunity 2: 561–572. 7540941 10.1016/1074-7613(95)90001-2

[40] Silva CL, Faccioli LH (1988) Tumor necrosis factor (cachectin) mediates induction of cachexia by cord factor from mycobacteria. Infect Immun 56: 3067–3071. 3053451 10.1128/iai.56.12.3067-3071.1988 PMC259702

[41] Andrade Junior DR, Santos SA, Castro I, Andrade DR (2008) Correlation between serum tumor necrosis factor alpha levels and clinical severity of tuberculosis. Braz J Infect Dis 12: 226–233. 18833408 10.1590/s1413-86702008000300013

[42] Macallan DC, McNurlan MA, Kurpad AV, de Souza G, Shetty PS, et al (1998) Whole body protein metabolism in human pulmonary tuberculosis and undernutrition: evidence for anabolic block in tuberculosis. Clin Sci (Lond) 94: 321–331. 9616267 10.1042/cs0940321

[43] Van Lettow M, Kumwenda JJ, Harries AD, Whalen CC, Taha TE, et al (2004) Malnutrition and the severity of lung disease in adults with pulmonary tuberculosis in Malawi. Int J Tuberc Lung Dis 8: 211–217. 15139450

[44] Douglas NM, Anstey NM, Buffet PA, Poespoprodjo JR, Yeo TW, et al (2012) The anaemia of Plasmodium vivax malaria. Malar J 11: 135. 22540175 10.1186/1475-2875-11-135 PMC3438072

